# Metal Concentrations in Tissues of Gadwall and Common Teal from Miankaleh and Gomishan International Wetlands, Iran

**DOI:** 10.1007/s12011-017-1237-2

**Published:** 2018-01-13

**Authors:** Mohammad-Hosein Sinkakarimi, Lukasz J. Binkowski, Mehdi Hassanpour, Ghasem Rajaei, Mohsen Ahmadpour, Jeffrey M. Levengood

**Affiliations:** 1 Department of Environmental Science, Faculty of Natural Resources and Environments, Malayer University, Malayer, Hamadan Iran; 2 Institute of Biology, Pedagogical University of Cracow, Krakow, Poland; 3 Department of Environment, Provincial Directorate of Environment Protection, Golestan, Iran; 4 Department of Environmental Science, Natural Resource and Environment Faculty, Birjand University, Birjand, Southern Khorasan Iran; 5 Department of Environmental Science, Faculty of Fisheries and Environmental Sciences, Gorgan University of Agricultural Sciences and Natural Resources, Gorgan, Iran; 6 Illinois Natural History Survey, University of Illinois at Urbana-Champaign, Champaign, USA

**Keywords:** Accumulation, Exposure, Poisoning, Biomonitoring, Ducks, Environment

## Abstract

Miankaleh and Gomishan International Wetlands are important wintering areas for waterbirds in the Caspian Sea region. Previous studies revealed increased exposure to metals in some species of waterbirds using these wetlands. In this study, we examined concentrations of cadmium (Cd), chromium (Cr), iron (Fe), lead (Pb), and zinc (Zn) in kidneys, liver, and pectoral muscle of wintering Gadwall (*Anas strepera*) and Common Teal (*Anas crecca*) collected in 2012. In addition, we measured concentrations of these elements in water and sediments from the collection sites. The genders differed in only one element/tissue combination, i.e., concentrations of Fe were greater in the livers of males. Concentrations of elements observed in Gadwall were generally higher than in Common Teal; only renal Cr and muscle Zn did not differ between species. Mean Cd concentrations in Gadwall exceeded background levels, reaching 1.94 μg/g ww in kidneys and 1.09 μg/g ww in liver. Similarly, Pb concentrations in Gadwall were also elevated (4.14 μg/g ww in kidneys, 3.22 μg/g ww in liver). Concentrations of other metals were within ranges commonly found in waterfowl. Concentrations of elements in the environment were elevated above background and comparable with the data obtained for this region by other scientists. However, these levels were deemed to not be great enough to pose an acute health risk to waterfowl. Given increased concentrations of some metals in duck tissues, further inquiry into the source of the exposure is needed for this area.

## Introduction

Many parts of the world have witnessed substantial industrial and urban development, which has both improved socioeconomic standards and threatened the ecosystem [43, 62]. The increased release of metals (such as cadmium (Cd), chromium (Cr), and lead (Pb)) associated with anthropogenic activity is one of the threats to ecosystem stability. These pollutants enter the environment through industrial processes, urban and suburban runoffs, agricultural practices, natural erosion, and geochemical cycles [15, 48]. Unlike many organic contaminants, metals cannot be degraded further so their toxic effects can be long lasting, and they accumulate in biota and can be transferred along the food chains [12, 13, 27]. Waterbirds have been extensively used in biomonitoring of environmental pollution as their ecology is generally known; they are long-lived, abundant, and may feed at different trophic levels [2, 19, 26, 35, 38, 58].

The extent of exposure of birds to metals depends on factors such as migration stage, migration route, characteristics of the environment, species and trophic level [1, 16, 60]. We examined metal concentrations in two species of dabbling ducks which have less contact with sediments than do diving ducks, so the potential exposure should not be high. Both species occupy similar habitats, but vary substantially in body size, as well as in ecology, migration pattern, and behavior. The influence of all of these factors is hard to predict, but our hypothesis was that the bigger species will have accumulated higher concentrations of elements studied (mainly due to a greater influence of diet). The sampling sites are within one of the most important waterbird wintering areas along the Caspian Sea [53]. There have been only a few published studies on environmental contaminants from this region, and none has examined metals in these species. We expected to find significantly increased environmental concentrations of metals, since some reports have identified oil extraction, transportation, inputs from cities, industries and agricultural wastes, and fishery practices as potential sources of pollution in this region [5, 28, 36, 52]. Previous studies have suggested elevated exposure of waterbirds to Cd and Pb in the area [4, 55]. Consequently, we examined the exposure of two species of ducks that we suspected may have elevated exposure to toxic metals in these important wetlands. In addition to more toxic elements that may interfere with avian physiology, we decided to also examine concentrations of selected essential elements that may be impacted through exposure to more toxic metals [22, 49].

The primary objectives of our study were (1) to examine concentrations of cadmium (Cd), chromium (Cr), iron (Fe), lead (Pb), and zinc (Zn) in tissues of Gadwall (*Anas strepera*) and Common Teal (*Anas crecca*) collected during wintering in south-coast of the Caspian Sea, (2) to examine concentrations of these metals in sediment and water samples from study wetlands, and (3) to determine if exposures were high enough to be a concern for avian health. We compared metal concentrations between species, sexes, and to reported literature in order to evaluate metal exposure of birds wintering on the southern coastal Caspian Sea. Additionally, we evaluated the relationships between metal concentrations in the tissues examined. Concentrations of metals within water and sediment samples were also compared to existing literature.

## Material and Methods

**Fig. 1:**
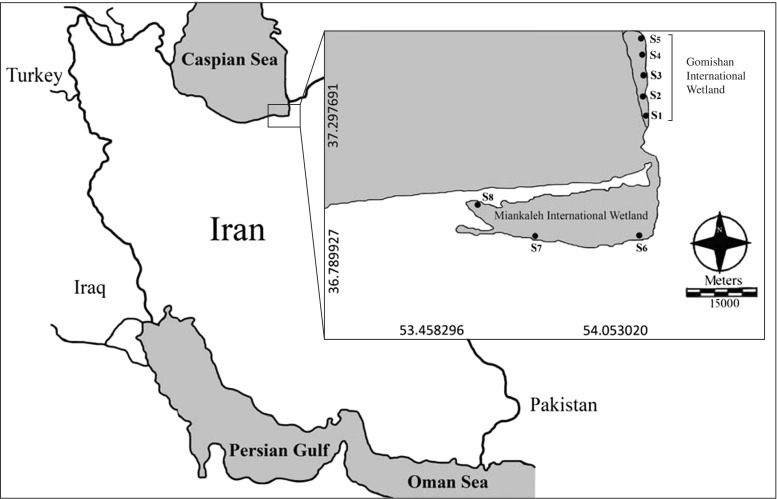
Sampling sites in Miankaleh and Gomishan International Wetlands

We examined 20 adult Gadwall and 20 adult Common Teal (equally divided between sexes) collected by hunters in February of 2012 from two sites in the southern Caspian Sea region: Miankaleh (66,933 ha) and Gomishan (20,000 ha) International Wetlands (Fig. 1). Waterfowl arrive to the study area between late October and early November and remain until late March and early April. Thus, prior to our collections, specimens occupied the study site for approximately 3 to 4 months. Bird carcasses were immediately transported to the laboratory (Golestan Provincial Office, Department of the Environment), and samples of kidneys, liver, and pectoral muscle were collected using sterile instruments to avoid external contamination. Since the hunters used size no. 4 Pb pellet ammunition, we used care to collect the samples in a way that avoided contamination from pellets. After the dissections, tissues were stored in chemically clean plastic bags and kept at − 20 °C until analysis.

Water samples were taken from three levels (surface, mid-column, and near-bottom, i.e., ca. 25 cm above the sediments), then pooled together in clean plastic bottles, acidified with HNO_3_ (10%, Merck, Germany) and transported to the laboratory. Sample sites for water collection were chosen based on aggregation of birds. We omitted sampling in places not commonly used by waterfowl.

After collection of water samples, sediment samples were collected with a grab sampler from the same eight sampling points on the study area (Fig. 1). Samples were transported to the laboratory, dried at 110 °C for 24 h, passed through a 0.063-mm plastic sieve, and stored in polyethylene bottles until chemical treatment.

### Laboratory Protocol

Approximately 5 g wet weight of each sample were placed in a porcelain crucible and dried at 135 °C for 2 h. Dry samples were transferred to a cool muffle furnace to be ashed overnight in the temperature slowly raising up to 450–500 °C. After cooling, 2 mL of ultrapure HNO_3_ (65%, Merck, Germany) were added and samples were dried on a hot plate. Samples were returned to the furnace and heated for an hour and then cooled. Next, 10 mL of 1 M HCL (37%, Merck, Germany) were added and solutions were heated to dissolve ash. Digested samples were filtered and diluted up to 25 ml with 1 M HCL [32].

Acid digestion of sediment samples was performed with 0.5 g of sample and 3 mL of mixture of concentrated HCl and HNO_3_ (volume ratio 3:1). Mineralization was carried out for 6 h at 90 °C, then 4 mL of concentrated HClO_4_ was added. Digested samples were filtered and diluted up to 25 mL with ultrapure water [23]. Water samples were only filtered prior to analysis with 0.42 mm filters (Whatman, UK).

Metal concentrations were determined using a graphite furnace atomic absorption spectrometer (GFS97, Thermo Electron, Cambridge, UK). Due to technical limitations, we were not able to measure Fe concentrations in water and sediments. The final concentrations in samples were expressed as micrograms per gram of wet weight (μg/g ww) for solid samples and as micrograms per liter (μg/L) for water samples. Detection limits were 0.004 μg/g for Cd, 0.03 μg/g for Cr, 0.05 μg/g for Fe, 0.001 μg/g for Pb, and 0.005 μg/g for Zn (for water per mL). Standard reference material (Mussel tissue—NIST SRM 2976) was used for the quality assurance and control. The precision was calculated as a relative standard deviation (RSD) of replicate samples of the prepared standard and was found to be less than 5%. All the recoveries ranged were from 95 to 105%.

### Statistical Analysis

Statistical analysis was performed with SPSS 18.0 (IBM) and Statistica 12 (StatSoft). Data was tested for the normal distribution (Shapiro–Wilk test) and variance homogeneity (Levene test). Since the data did not fulfill the assumption of parametric analyses, we ran robust main effects ANOVA on ranks to test the differences in metal concentrations in birds between sexes and species [51]. We pooled tissue concentrations between sampling sites due to their close proximity to each other and likelihood of birds traveling and foraging throughout the entire study area. The comparisons of metal concentrations in water and sediments between sampling sites were done with Mann–Whitney test.

The relationships between variables tested were evaluated with Pearson coefficients. We decided to run correlation analysis with the pooled data from both species to ensure more reliability and precision of the inference. Only the statistically significant correlations with Pearson *r* higher than 0.6 were discussed. Significance level was 0.05 for all the analyses.

## Results and Discussion

**Fig. 2:**
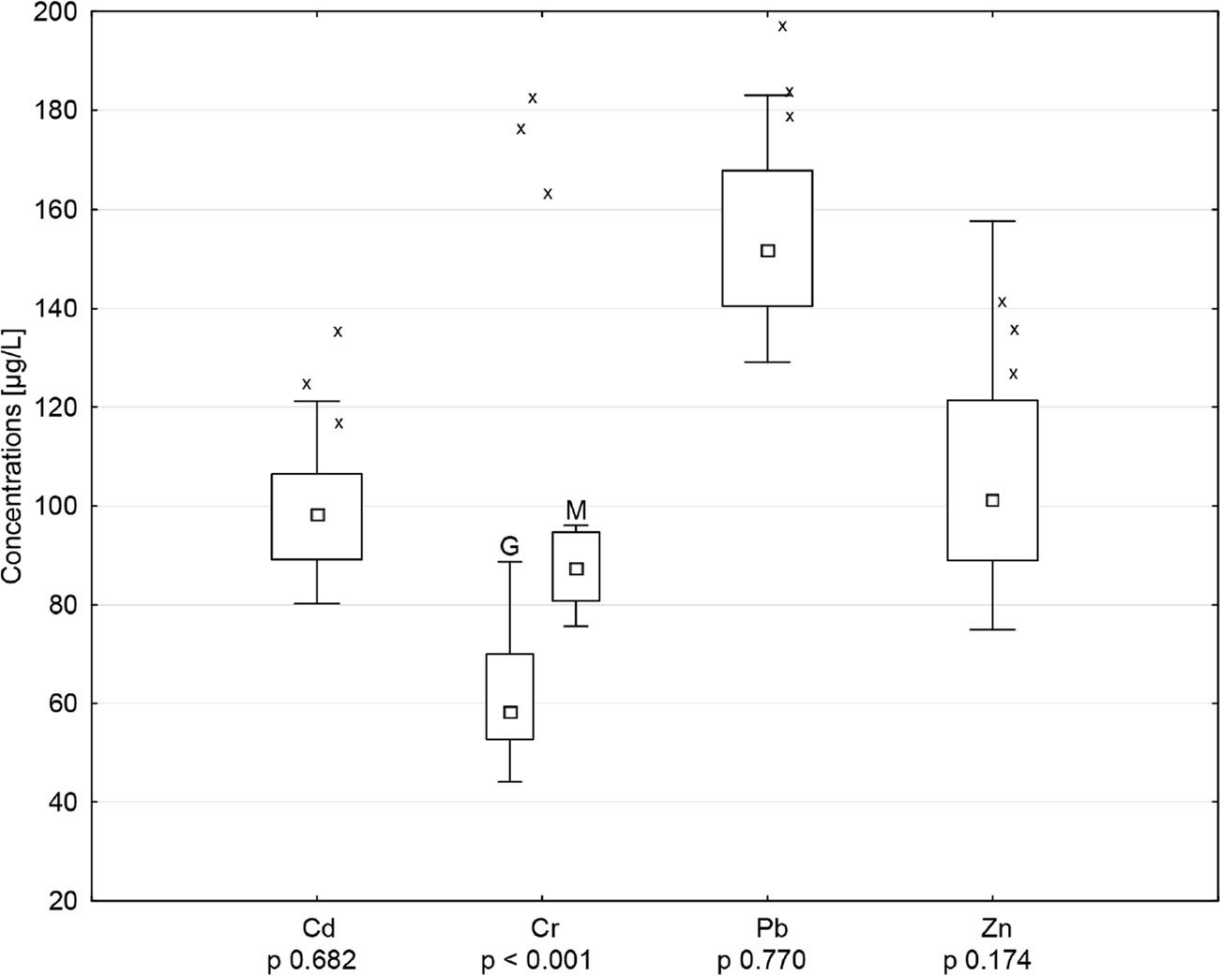
Metal concentrations (medians, quartiles, and ranges) in water with statistical comparison between Miankaleh (M) and Gomishan (G) areas. The data marked with crosses was obtained for samples from S6 sampling point. Since the results marked deviated significantly from the medians, they were excluded from the statistical comparison

**Fig. 3:**
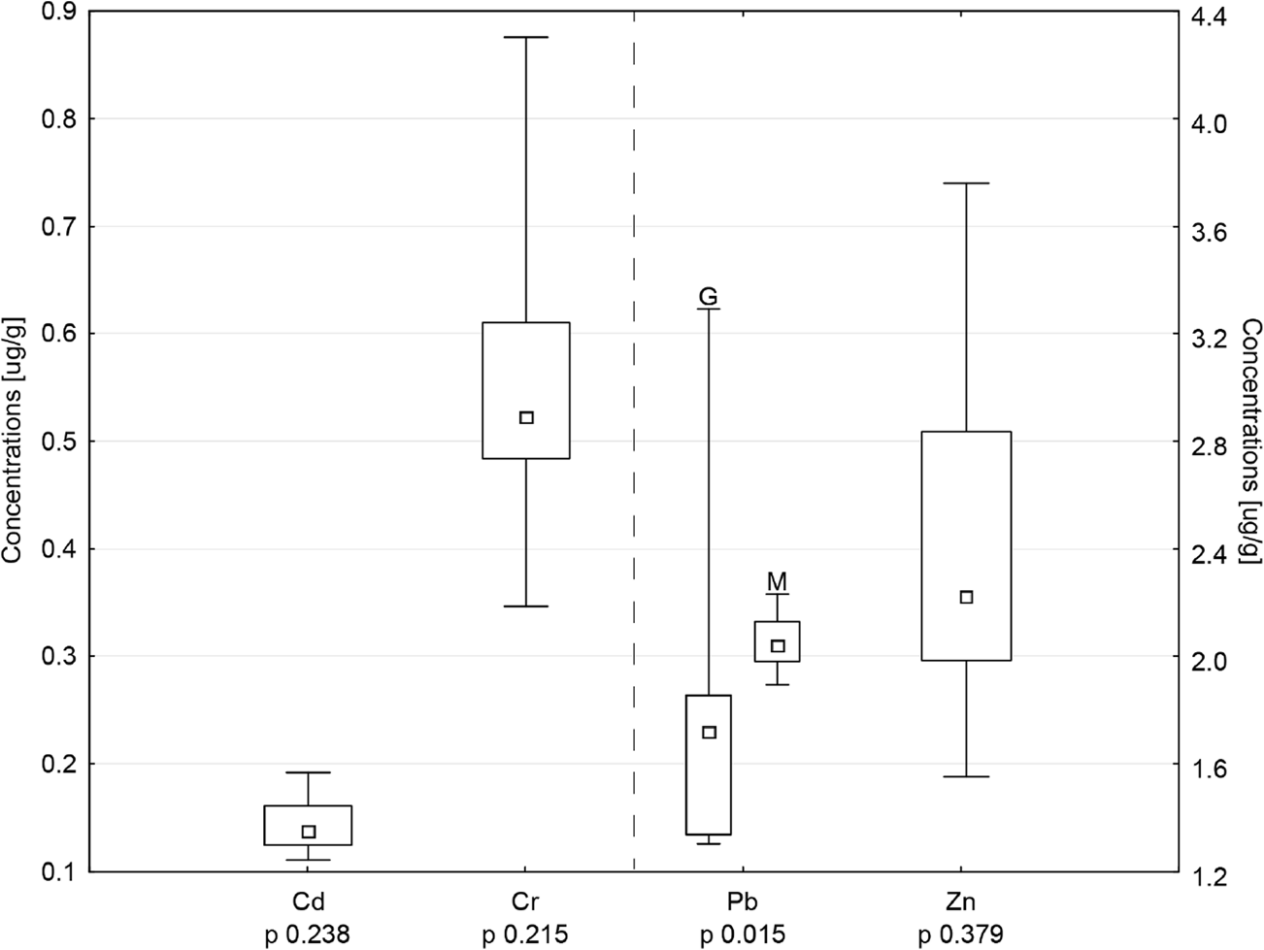
Metal concentrations in sediments (medians, quartiles, and ranges) with statistical comparison between Miankaleh (M) and Gomishan (G) areas

Concentrations in environmental samples differed statistically between areas only in the case of Cr in water (median 87.5 μg/L on Miankaleh area vs 58.2 μg/L on Gomishan area) and Pb in sediments (median 1.7 μg/g on Miankaleh area vs 2.1 μg/g on Gomishan area). In water, the highest concentrations were noted for Pb (median 151.7 μg/L) and the lowest for Cr (Fig. 2). Pb concentrations were significantly lower than those producing toxic effects in duck embryos (2.9 mg/L; Kertesz et al. [37]). However, concentrations of elements were elevated in comparison to clean sea water (referenced to Cd 0.9, Cr 0.3, Pb 0.6, and Zn 39 μg/L), which probably is a consequence of input from local industry and agriculture [34]. We concluded that water on both sites has increased concentrations of elements studied, but it is not heavily polluted and should not pose a risk for birds. In the case of sediments we examined, the highest concentrations were noted for Cr and the lowest for Cd (Fig. 3). A similar distribution (Cr < Pb < Cd) was found in the Varano Lagoon (Adriatic Sea) in Italy, but the concentrations there were visibly higher (3.31, 1.60, and 0.19 μg/g consecutively; [57]). Similarly, Pb concentrations found in Baltic Sea sediments (Poland) were significantly higher than those from Miankaleh and Gomishan area [61]. Levengood and Skowron [42] found an increased accumulation of Cd in kidneys of mallards exposed to sediments with considerably greater Cd concentrations (173 μg/g) that those noted in our study. The ingestion of sediments in waterfowl is usually on the level of 2–3% (in some species reaches 22%), and in the case of higher environmental concentrations, sediment ingestion can be the principal route of exposure to environmental contaminants [41, 7–10]. In respect of our results, we concluded that the levels found in Miankaleh and Gomishan area would likely not pose an acute health risk to the health of waterbirds utilizing the wetlands, but still the chronic exposure is possible.

Except for Fe concentrations in liver, of which males had higher concentrations (Gadwall 390.51 vs 265.50 μg/g; Common Teal 210.06 vs 149.63 μg/g), we found no significant differences in metal concentrations between males and females (Table 1). Previous literature has reported conflicting results for metal accumulation between sexes in waterbirds. Metal concentrations did not differ between sexes in the liver and kidneys of Canvasback (*Aythya valisineria*) [24], liver and blood of Mallard (*Anas platyrhynchos*), Pintail (*Anas acuta*), Shoveler (*Anas clypeata*), and Tufted Duck (*Aythya fuligula*) [14, 50]. In contrast, significant differences in Cd, Cu, Mn, Pb, and Zn concentrations between males and females were noted in liver of Scaup (*Aythya marila*) [29], kidneys, liver, and feathers of Common Coot (*Fulica atra*) and Mallard [45] and kidneys, liver, and muscles of Pochard (*Aythya ferina*) [55]. It is likely that differences in foraging habits and physiology between males and females, coupled with season of collection and distribution of elements among habitats, drive differences in exposures between the genders.

**Table 1:** Statistical comparison (*p* values) between sexes of Gadwall (*n* = 20) and Common Teal (*n* = 20). Main effects factorial ANOVA on ranks

Tissue	Cd	Cr	Fe	Pb	Zn
Kidneys	0.628	0.289	0.417	0.184	0.355
Liver	0.978	0.482	0.045	0.366	0.235
Muscle	0.434	0.921	0.154	0.135	0.833

Underline indicates statistically significant differences

Muscle samples contained the lowest concentrations of elements among the tissues, with Cr having the lowest (0.08 μg/g in Common teal) and Fe the highest median (65.32 μg/g in Gadwall) concentrations. The kidneys contained the highest concentrations of Cd (1.94 μg/g in Gadwall) and Pb (4.14 μg/g in Gadwall), whereas Fe (379.54 μg/g in Gadwall) and Zn (20.00 μg/g in Gadwall) concentrations were greatest in liver. Chromium concentrations were similar in all the tissues. With the exception of several cases (Cd in liver, Cr in kidneys and liver, Pb in liver, and Zn in pectoral muscles), concentrations of metals were significantly higher in Gadwall than in Common Teal (Table 2). Both species of ducks feed on invertebrates and plants on the same wetlands, but they may partition themselves into different microhabitats and forage at different locations and depths within a wetland, which can result in different exposure and accumulation [3, 44]. Moreover, Common Teal is smaller than Gadwall, and consumes fewer and smaller food items, which probably influences their overall metal exposure [20].

**Table 2:** Metal concentrations in tissues of Gadwall (*n* = 20) and Common Teal (*n* = 20) collected on southeastern Caspian Sea (μg/g ww) with statistical comparison between species (sexes were polled basing on the results of Table 1). Main effects factorial ANOVA on ranks (*p* values presented)

		Cd	Cr	Fe	Pb	Zn
Gadwall						
Kidneys	Median	1.94	0.25	153.06	4.14	14.30
	Min-max	0.87–2.70	0.11–0.55	101.04–310.52	2.40–6.66	7.88–24.33
Liver	Median	1.09	0.33	379.54	3.22	20.00
	Min-max	0.51–1.91	0.19–0.62	170.30–611.38	1.56–5.66	15.22–36.16
Muscle	Median	0.35	0.16	65.32	0.95	8.14
	Min-max	0.17–0.91	0.09–0.51	29.31–110.08	0.55–1.30	3.49–13.53
Common Teal						
Kidneys	Median	0.85	0.23	91.14	1.22	9.38
	Min-max	0.4–2.17	0.08–0.42	70.46–214.30	0.65–2.61	4.39–21.36
Liver	Median	0.83	0.17	178.52	1.08	11.11
	Min-max	0.4–1.65	0.05–0.31	98.33–420.53	0.55–2.09	5.62–27.15
Muscle	Median	0.10	0.08	31.82	0.27	8.72
	Min-max	0.04–0.35	0.03–0.14	16.70–93.32	0.08–0.96	3.79–13.39
Kidneys	Species *p*	< 0.001	0.567	< 0.001	< 0.001	0.002
Liver	Species *p*	0.978	0.482	0.045	0.366	0.235
Muscle	Species *p*	< 0.001	< 0.001	0.001	< 0.001	0.495

Underlines indicate statistically significant differences

Kidneys are the primary repository tissue for Cd, and along with the liver, accumulate more than 70% of total body burden in birds [54]. Background Cd levels in kidneys and liver are estimated as < 8 and < 3 μg/g dw, respectively (recalculated to ww ≈ 1.9 and 1 μg/g; [11]) and higher concentrations are a consequence of increased exposure [54]. This phenomenon was observed among more than half of Gadwalls studied, whose median concentrations exceeded the background levels. Median concentrations in kidneys and livers of Common Teal were lower than in Gadwall, although some individuals exceeded background levels also (Table 2). Cadmium concentrations that we observed in the kidneys and liver of Gadwall were higher than noted for ecologically similar species on other areas such as western Iran, southern Poland, and eastern Croatia [16, 25, 45]. However, even higher concentrations were reported by Hassanpour et al. [31] and Sinkakarimi et al. [55] in kidneys and liver of Common Coot (2.11 and 2.22 μg/g ww) and Pochard (2.16 and 1.63 μg/g ww) collected from the same wetlands as our specimens, confirming that waterbirds using Miankaleh and Gomishan International Wetlands are exposed to increased concentrations of Cd. The type of Cd exposure (i.e., acute vs. chronic) may be evaluated on the basis of the ratio of liver to kidney concentrations. When this factor exceeds 1, it is considered acute exposure, whereas a ratio below 1 indicates chronic exposure to low levels of Cd [54]. The ratios in the birds we examined ranged from 0.56 for Gadwall to 0.97 for Common Teal, indicating chronic Cd exposure, as has been reported in a number of avian species [6, 33, 40]. Based on the results of concentrations in the environment (Figs. 2 and 3), we suspect water as the source of chronic exposure to Cd. Although the concentrations were not acutely toxic, with longer exposure time, they may accumulate in ducks. There is also a possibility that the ducks may come to the wintering areas with elevated levels in tissues as the consequence of the exposure on breeding sites or migration stopovers. This could explain the different concentrations observed in these species, as they breed in different regions [21].

Gadwall and Common Teal, along with other species of waterfowl, are potentially threatened with Pb poisoning resulting from ingestion of hunting ammunition and fishing sinkers [18]. During foraging, birds may ingest Pb pellets as grit or in mistake for small seeds, which dissolve in the digestive tract causing toxicosis ([17]). Gadwall and Common Teal often forage by probing the sediment for invertebrates and seeds, thus they are likely to encounter Pb shot both on and under the sediment surface. Gadwalls contained mean concentrations of Pb in kidneys (4.14 μg/g) and liver (3.22 μg/g) that were higher than the lowest proposed threshold for Pb poisoning (3.0 and 1.5 μg/g ww, consecutively; [30]), and in fact, concentrations observed in all livers were above this level (Table 2). Only three Common Teals revealed higher concentrations in liver than toxicity thresholds. Thus, Gadwall had greater exposure to Pb than Common Teal, and they may have ingested locally expelled Pb pellets or fishing sinkers since the concentrations we observed were higher than in other waterfowl species from Iran, Poland, Croatia, and Spain; [17, 25, 45, 59]). The increased exposure to Pb particles was also suspected in other species from the same area in another study in which increased Pb concentrations were observed in livers of Pochards (2.36 μg/g) and Mallards (1.16 μg/g) [55].

Essential elements such as Cr, Fe, and Zn are necessary for metabolism and normal tissue levels are maintained by homeostatic mechanisms [48]. The concentrations we observed in kidneys and livers of Gadwall and Common Teal in this study were within the ranges commonly found in other waterfowl [39, 45–47].

We noted a significant (*p* < 0.05) relationship between Fe concentrations in kidneys and liver (Pearson *r* = 0.66) as well as between Pb and Cd in kidneys (*r* = 0.72), Pb in muscles and liver (*r* = 0.71), Pb with Cr in muscles (r = 0.66), Pb in muscles and kidneys (0.66), Pb in kidneys with Cd in muscles (*r* = 0.65), and Pb in muscles with Zn in liver (*r* = 0.60). No correlation between concentrations of Fe and any other element was found, suggesting that there are no impairments in physiology due to exposure to metals studied. Positive correlations between concentrations of elements as Cd, Cr, Pb, and Zn in tissues of birds studied could result from a mutual source of metals in the environment, since most pollution inputs (including transport, industry, and agriculture common in the area studied) often include a variety of elements [42, 56].

## Conclusions

With a few exceptions, the tissues of Gadwall contained greater concentrations of elements than those of Common Teal. These species comparisons may relate to differing microhabitat, foraging locations, and water/sediment depths, or the amount of food consumed daily. Gadwall may have been chronically exposed to Cd. Our findings agree with previous studies assessing metal accumulation among waterbirds utilizing Miankaleh and Gomishan International Wetlands, which found increased exposure to Cd and Pb. The levels found in water and sediments of both wetlands were above background though not high enough to pose a clear risk to the health of birds.
